# ICTV Virus Taxonomy Profile: *Caulimoviridae*

**DOI:** 10.1099/jgv.0.001497

**Published:** 2020-09-17

**Authors:** Pierre-Yves Teycheney, Andrew D. W. Geering, Idranil Dasgupta, Roger Hull, Jan F. Kreuze, Ben Lockhart, Emmanuelle Muller, Neil Olszewski, Hanu Pappu, Mikhail M. Pooggin, Katja R. Richert-Pöggeler, James E. Schoelz, Susan Seal, Livia Stavolone, Marie Umber, ICTV Report Consortium

**Affiliations:** ^1^​ CIRAD, UMR AGAP, F-97130 Capesterre-Belle-Eau, Guadeloupe, France; ^2^​ AGAP, Univ Montpellier, CIRAD, INRAE, Montpellier SupAgro, Montpellier, France; ^3^​ Queensland Alliance for Agriculture and Food Innovation, The University of Queensland, GPO Box 267, Brisbane, Queensland 4001, Australia; ^4^​ Department of Plant Molecular Biology, University of Delhi South Campus, New Delhi 110021, India; ^5^​ Child Okeford, Blandford Forum, Dorset, UK; ^6^​ International Potato Center (CIP), Apartado 1558, Lima 12, Peru; ^7^​ Department of Plant Pathology, University of Minnesota, St. Paul, Minnesota, USA; ^8^​ CIRAD, UMR BGPI, F-34398 Montpellier, France; ^9^​ BGPI, Univ Montpellier, CIRAD, INRAE, Montpellier SupAgro, Montpellier, France; ^10^​ Department of Plant Biology, University of Minnesota, Minneapolis, Minnesota, USA; ^11^​ Department of Plant Pathology, Washington State University, Pullman, Washington, USA; ^12^​ INRA, UMR BGPI, F-34398 Montpellier, France; ^13^​ Julius Kühn-Institut, Institute for Epidemiology and Pathogen Diagnostics, Braunschweig, Germany; ^14^​ Division of Plant Sciences, University of Missouri, Columbia, Missouri, USA; ^15^​ Natural Resources Institute, University of Greenwich, Chatham, Kent ME4 4TB, UK; ^16^​ Consiglio Nazionale delle Ricerche, Istituto per la Protezione Sostenibile delle Piante, Bari, Italy; ^17^​ International Institute of Tropical Agriculture, Ibadan, Nigeria; ^18^​ INRAE, UR ASTRO, F-97170, Petit-Bourg, Guadeloupe, France

**Keywords:** *Caulimoviridae*, ICTV Report, taxonomy

## Abstract

*Caulimoviridae* is a family of non-enveloped reverse-transcribing plant viruses with non-covalently closed circular dsDNA genomes of 7.1–9.8 kbp in the order *Ortervirales*. They infect a wide range of monocots and dicots. Some viruses cause economically important diseases of tropical and subtropical crops. Transmission occurs through insect vectors (aphids, mealybugs, leafhoppers, lace bugs) and grafting. Activation of infectious endogenous viral elements occurs in *Musa balbisiana*, *Petunia hybrida* and *Nicotiana edwardsonii*. However, most endogenous caulimovirids are not infectious. This is a summary of the International Committee on Taxonomy of Viruses (ICTV) Report on the family *Caulimoviridae,* which is available at ictv.global/report/caulimoviridae.

## Virion

**Fig. 1.**
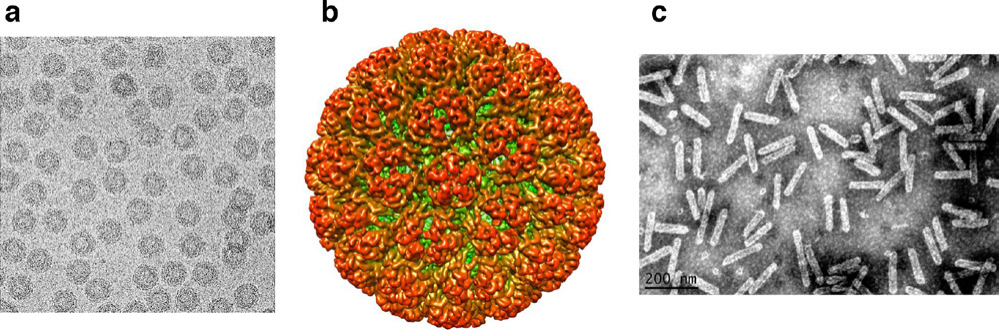
Negative-contrast electron micrographs of virions of (a) cauliflower mosaic virus and (c) banana streak MY virus. (b) Tridimensional reconstruction of the cauliflower mosaic virus particle (images courtesy of Patrick Bron and Andrew D.W. Geering).

Virions are either isometric of 45–52 nm in diameter or, in the case of members of the genera *Badnavirus* and *Tungrovirus,* bacilliform particles of 30 nm × 60–900 nm (Table 1, Fig. 1). Virion sedimentation coefficient (S_20,w_) is 200–220 S; density in CsCl is 1.37 g cm^−3^. No envelope is present.

**Table 1.** Characteristics of members of the family *Caulimoviridae*

Typical member:	cauliflower mosaic virus-Cabb-S (V00141), species *Cauliflower mosaic virus*, genus *Caulimovirus*
Virion	Non-enveloped, isometric or bacilliform with a single-core capsid protein
Genome	7.1–9.8 kbp of non-covalently closed circular dsDNA with discontinuities in both genome strands at specific places
Replication	Cytoplasmic via reverse transcription of pregenomic RNA by viral reverse transcriptase. Terminally redundant pregenomic RNA is transcribed in the nucleus from repaired, covalently closed circular dsDNA by host DNA-directed RNA polymerase II
Translation	From capped and polyadenylated pregenomic RNA; in some viruses from subgenomic RNA and spliced versions of pregenomic RNA
Host range	Plants (monocots and dicots); some are transmitted by insects
Taxonomy	Realm *Riboviria,* kingdom *Pararnavirae,* phylum *Artverviricota,* class *Revtraviricetes,* order *Ortervirales,* multiple genera including >80 species

## Genome

**Fig. 2.**
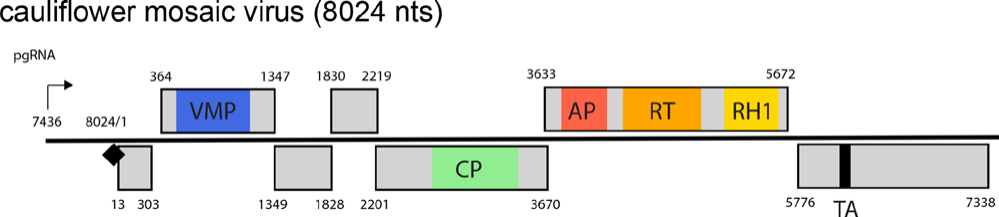
Caulimovirus genome linearised at the pregenomic RNA transcription start site (black arrow), numbered from the Met-tRNA primer binding site (black diamond). ORFs (light grey) include domains for the viral movement protein (VMP, blue), coat protein conserved C-terminus (CP, green), retropepsin (pepsin-like aspartic protease, AP, red), reverse transcriptase (RT, orange), RNase H1 (RH1, yellow), and translation transactivator (TA, black).

Virions contain a single molecule of non-covalently closed circular dsDNA of 7.1–9.8 kbp [1, 2] with discontinuities at specific sites in the negative-sense (one) and positive-sense strand (one to three). Genomes contain 1–8 ORFs encoding 5–6 conserved protein domains (Fig. 2), depending on the genus.

## Replication

Following entry into the cell, the virion is targeted to the nucleus by a nuclear localization signal in the N-terminus of the capsid protein. Discontinuities in the genome are sealed to give supercoiled DNA, which associates with histone proteins to form mini-chromosomes in the nucleus. These are transcribed by host DNA-directed RNA polymerase II to give a greater-than-genome length transcript (35S or 34S RNA) that has a terminal redundancy of 35 to 270 nt. This transcript (pregenomic RNA) serves as a template for reverse transcription to give the negative-sense strand DNA and as a polycistronic mRNA for expression of at least some of the ORFs [3].

Unlike retroviruses, the episomal replication cycle does not involve an integration phase [4–6]. Negative-sense strand DNA synthesis is primed by host cytosolic tRNA^met^. Synthesis of both strands is performed by the viral reverse transcriptase and RNase H1. RNase H1-resistant polypurine stretches serve as primer for positive-sense DNA synthesis. The site-specific discontinuities are at the priming sites for both negative- and positive-sense strand DNA synthesis and are made by the oncoming strand displacing the existing strand for a short distance and not ligating to form a closed circle [2].

## Taxonomy

Current taxonomy: ictv.global/report/caulimoviridae. Members of the genera *Badnavirus* and *Tungrovirus* have bacilliform virions whereas members of the genera *Caulimovirus*, *Cavemovirus*, *Petuvirus*, *Rosadnavirus*, *Solendovirus* and *Soymovirus* have isometric virions. The number of ORFs ranges between one (petuviruses and vacciniviruses), three or more (badnaviruses), four (cavemoviruses, dioscoviruses, solendoviruses and tungroviruses), seven (caulimoviruses), seven or eight (soymoviruses) and eight (rosadnaviruses). Insect-mediated transmission has been reported for badnaviruses, caulimoviruses and tungroviruses. Infectious endogenous viral elements (EVEs) have been reported for several banana streak viruses (*Badnavirus*), petunia vein clearing virus (*Petuvirus*) and tobacco vein clearing virus (*Solendovirus*).

## Resources

Current ICTV Report on the family *Caulimoviridae*: ictv.global/report/caulimoviridae
